# Nigrostriatal degeneration determines dynamics of glial inflammatory and phagocytic activity

**DOI:** 10.1186/s12974-024-03091-x

**Published:** 2024-04-12

**Authors:** Leyre Ayerra, Miguel Angel Abellanas, Leyre Basurco, Ibon Tamayo, Enrique Conde, Adriana Tavira, Amaya Trigo, Clara Vidaurre, Amaia Vilas, Patxi San Martin-Uriz, Esther Luquin, Pedro Clavero, Elisa Mengual, Sandra Hervás-Stubbs, Maria S. Aymerich

**Affiliations:** 1 Facultad de Ciencias, Departamento de Bioquímica y Genética, Universidad de Navarra, Pamplona, Spain; 2 CIMA-Universidad de Navarra, Pamplona, España; 3 Facultad de Medicina, Departamento de Patología, Anatomía y Fisiología, Universidad de Navarra, Pamplona, Spain; 4 Servicio de Neurología, Hospital Universitario de Navarra, Pamplona, Spain; 5 IdiSNA, Instituto de Investigación Sanitaria de Navarra, Pamplona, Spain

**Keywords:** Parkinson´s disease, Microglia, Neurodegeneration, Phagocytosis

## Abstract

Glial cells are key players in the initiation of innate immunity in neurodegeneration. Upon damage, they switch their basal activation state and acquire new functions in a context and time-dependent manner. Since modulation of neuroinflammation is becoming an interesting approach for the treatment of neurodegenerative diseases, it is crucial to understand the specific contribution of these cells to the inflammatory reaction and to select experimental models that recapitulate what occurs in the human disease. Previously, we have characterized a region-specific activation pattern of CD11b^+^ cells and astrocytes in the α-synuclein overexpression mouse model of Parkinson´s disease (PD). In this study we hypothesized that the time and the intensity of dopaminergic neuronal death would promote different glial activation states. Dopaminergic degeneration was induced with two administration regimens of the neurotoxin 1-methyl-4-phenyl-1,2,3,6-tetrahydropyridine (MPTP), subacute (sMPTP) and chronic (cMPTP). Our results show that in the sMPTP mouse model, the pro-inflammatory phenotype of striatal CD11b^+^ cells was counteracted by an anti-inflammatory astrocytic profile. In the midbrain the roles were inverted, CD11b^+^ cells exhibited an anti-inflammatory profile and astrocytes were pro-inflammatory. The overall response generated resulted in decreased CD4 T cell infiltration in both regions. Chronic MPTP exposure resulted in a mild and prolonged neuronal degeneration that generated a pro-inflammatory response and increased CD4 T cell infiltration in both regions. At the onset of the neurodegenerative process, microglia and astrocytes cooperated in the removal of dopaminergic terminals. With time, only microglia maintained the phagocytic activity. In the ventral midbrain, astrocytes were the main phagocytic mediators at early stages of degeneration while microglia were the major phagocytic cells in the chronic state. In this scenario, we questioned which activation pattern recapitulates better the features of glial activation in PD. Glial activation in the cMPTP mouse model reflects many pathways of their corresponding counterparts in the human brain with advanced PD. Altogether, our results point toward a context-dependent cooperativity of microglia/myeloid cells and astrocytes in response to neuronal damage and the relevance of selecting the right experimental models for the study of neuroinflammation.

**Graphical abstract:**

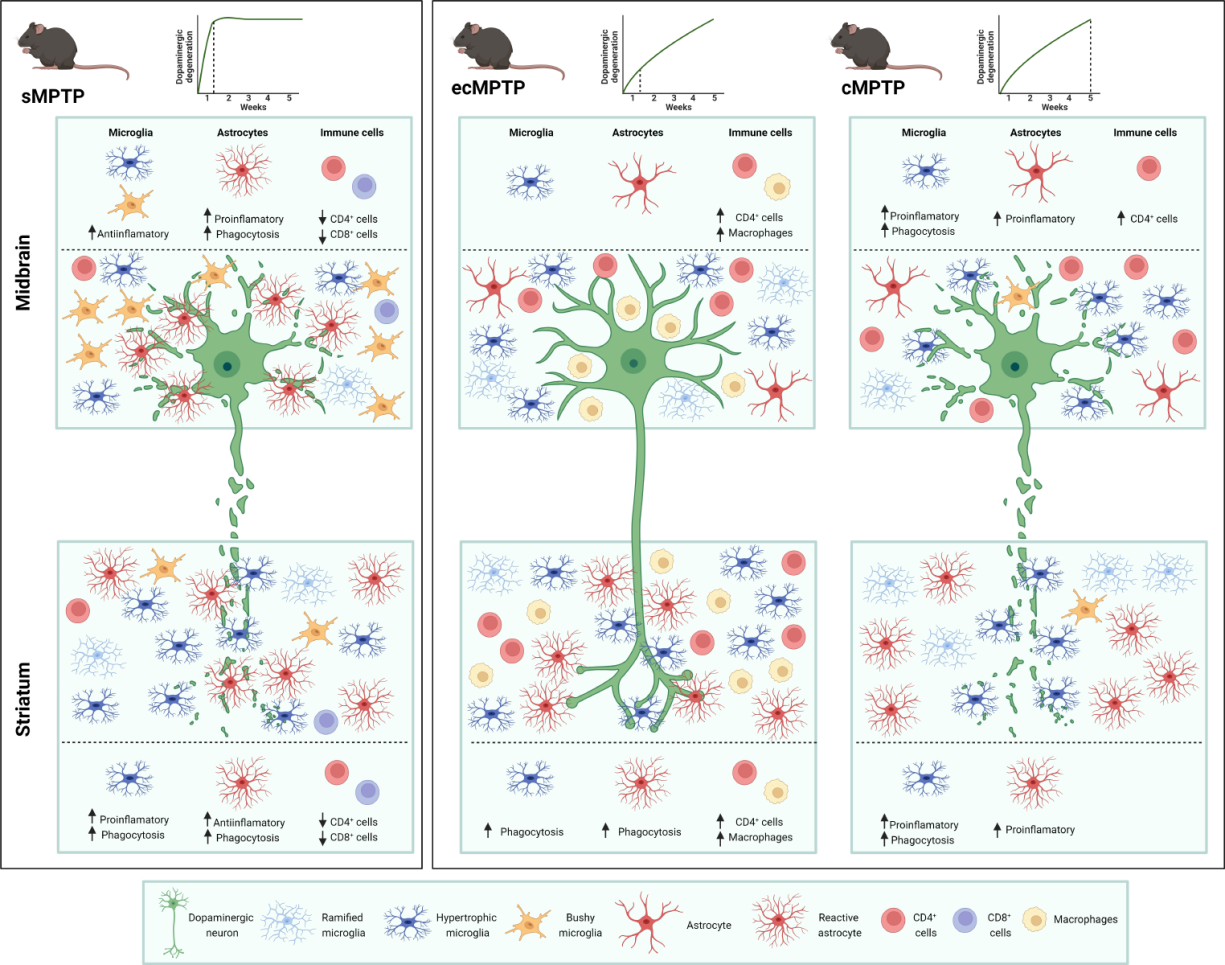

**Supplementary Information:**

The online version contains supplementary material available at 10.1186/s12974-024-03091-x.

## Background

Glial cells are key players in response to brain damage. During neurodegeneration, the beneficial or detrimental effect exerted by these cells over neurons depends on time, duration, context and type of stimulus. Microglia and astrocytes express toll-like receptors (TLRs) that under pathological conditions can be activated by non-infectious ligands to initiate innate immune responses [1–4]. A switch in their basal activation state generates a pronounced transformation characterized by morphological changes, gene expression, cytokine release or acquisition of a phagocytic phenotype. Throughout these processes, they have the ability to restrict or propagate inflammatory responses in the brain parenchyma [5].

The transcriptional profile of microglia shows spatial diversity, and non-uniform and region-dependent aging, being the immunoregulatory and metabolic pathways the major sources of heterogeneity [6]. Under steady-state conditions, microglia in the ventral tegmental area (VTA) and nucleus accumbens (NAc) share the immune gene profile and differ in genes involved in cell metabolism and phagocytosis that were downregulated and upregulated, respectively, in VTA microglia [7]. The ventral midbrain presents a high immune-alert state, not observed in the striatum, that includes two unique microglial subpopulations, one with antigen-presenting properties and another one expressing TLR4, and a higher proportion of infiltrated T cells [8]. In these regions, astrocytes play a relevant role in the maintenance of dopaminergic systems. They contribute to monoamine metabolism by taking up extrasynaptic dopamine [9] and by releasing neurotrophic factors necessary for survival of dopaminergic neurons [10–13]. Loss of homeostasis caused by dopamine neuron impairment will modify local microenvironment driving glial cells to the acquisition of new context-specific functions, in detriment of their physiological activities.

The first evidence for the involvement of microglial cells in Parkinson´s disease (PD) pathogenesis arise after the discovery of human leukocyte antigen-DR isotype (HLA-DR)-positive microglia/macrophages in post-mortem substantia nigra pars compacta (SNpc) [14]. Since then, the expression of different pro-inflammatory mediators in microglia, including tumor necrosis factor alpha (TNFα), interleukin 1 beta (IL1β), IL6, interferon-γ (IFNγ), nitric oxide synthase and cyclo-oxygenase, has been reported [15–19]. Genome-wide association studies (GWAS) have identified several single-nucleotide polymorphisms associated with PD risk, many of them linked to inflammation [20, 21] and a conveying risk of both, PD and autoimmune diseases [22, 23]. A higher incidence of PD is observed in patients with inflammatory bowel disease and it can be reduced with early anti-TNF therapy [24]. Astrocytes derived from induced pluripotent stem cells of PD patients show an exacerbated pro-inflammatory tone under inflammatory conditions [25]. A single-cell sequencing study of midbrain nuclei shows a pro-inflammatory profile and an increase in microglia and astrocytes fractions in this region, reinforcing the relevance of neuroinflammation in idiopathic PD [26]. Reactive glial cells exhibit a bidirectional communication in which microglia can trigger the induction of neurotoxic reactive astrocytes with reduced phagocytic capacity [27] and reactive astrocytes can prevent secretion of pro-inflammatory mediators by microglia [28]. Although a growing body of evidence suggests that glial cells likely contribute to PD pathogenesis, the reactivity of microglia and astrocytes in PD is still poorly understood. Microglial response occurs early in PD and might contribute to disease progression. However, whether the immune response is deleterious in each state of the disease remains to be defined [29].

In our previous studies using an adeno-associated viral system to overexpress α-synuclein in the SNpc, we observed that, during the active phase of degeneration, reactive glial cells showed a complementary activation and region-dependent phenotype. In the ventral midbrain, myeloid cells exhibited a phagocytic and anti-inflammatory profile, while astrocytes were the main contributors to the pro-inflammatory reaction. By contrast, myeloid cells expressed a pro-inflammatory profile in the striatum [30]. Since modulation of the inflammatory response is an interesting alternative for the treatment of dopaminergic neurodegeneration, we questioned whether the mechanism and the dynamics of dopamine neuronal death could affect glial reactivity and the inflammatory reaction. In this study, dopaminergic degeneration was induced with the neurotoxin 1-methyl-4-phenyl-1,2,3,6-tetrahydropyridine (MPTP). Considering that the onset and progression of disease represent distinct phases of the neurodegenerative process, we have selected two MPTP intoxication regimens, the widely used subacute (sMPTP) and the chronic (cMPTP) pattern, to analyze microglial/myeloid cell and astrocyte reactivity in the ventral midbrain and in the striatum. The transcriptomic profile obtained from the glial cells of each experimental model has been compared to their human counterparts [26].

## Materials and methods

### Animals

Adult male 3 or 4-month-old C57BL6J (20–30 g) mice were obtained from Envigo (Barcelona, Spain). Mice were housed at 21 °C on a 12 h light/dark cycle in a humidity-controlled environment with *ad libitum* access to food with standard rodent pellet diet (Envigo) and free access to water. All procedures involving animals were carried out in accordance with the Spanish National Research Council’s guide for the care and use of laboratory animals. The experimental designs were approved by the Ethical Committee for Animal Testing at the University of Navarra (Ref. 109 − 18).

### MPTP intoxication

Different patterns of MPTP (MedChemExpress, Shanghai, China) intoxication were used. In the subacute MPTP (sMPTP) regimen, 30 mg/kg of MPTP dissolved in saline was administered intraperitoneal (i.p.) for 5 consecutive days. Control mice for this group received saline at the same time. In the chronic MPTP (cMPTP) regimen, mice received 10 i.p. injections of MPTP (20 mg/kg in saline) together with probenecid (250 mg/kg in saline; Life Technologies, Eugene, OR, USA) to retard the renal clearance of toxic metabolites of MPTP. Both compounds were injected twice a week for 5 weeks in two consecutive injections separated 30–60 min. To generate an early stage of the chronic MPTP mouse model (ecMPTP), mice received 3 i.p. injections of MPTP (20 mg/kg) plus probenecid (250 mg/kg). Control mice were prepared administering only the probenecid injection at the corresponding days.

### Motor behavior

Motor behavior was analyzed in the rotarod, pole and bar test under low light conditions. The pole and the bar test were performed 4 h after the last MPTP injection and the rotarod 16 h later. In the pole test, animals were placed heading up on the top of a vertical wooden pole of 50 cm height and 1 cm in diameter covered with a bandage. Animals were pre-trained before the first MPTP dose until they were able to turn their head down and to descend from the pole in less than 5 s. The average time to turn the head down and to descend the pole was measured in two consecutive trials with a resting time of 15 min between them. The results represent the average of the two trials. In the bar test, animals were placed with the forepaws on a bar parallel to the ground at 4 cm height. The average time to move the forepaws to the ground was measured in three consecutive trials. In the rotarod (LE8200, Panlab, Barcelona, Spain) test, animals were placed over a rod programmed to increase speed from 4 r.p.m to 40 r.p.m. in 5 min. All animals were pre-trained before MPTP intoxication for 2 consecutive days until they were able to remain in the rod for a minimum of 1 min. The average time each mouse stayed on the rod was recorded in two trials with a resting time of 30 min between them.

### Preparation of brain cell suspensions

Adult mice were anesthetized with i.p. ketamine/xylazine and perfused transcardially with ice-cold phosphate-buffered saline (PBS, Lonza, Verviers, Belgium). The striatum and midbrain were dissected on ice and digested with a collagenase D mix (400 units/mL, Roche, Mannheim, Germany) or a papain mix (2 mg/mL, Roche, Mannheim, Germany) containing DNase-I (50 µg/mL, Sigma-Aldrich) in Dulbecco’s PBS (DPBS, Lonza, Walkersville, MO, USA). The tissue was digested at 37 °C under rotation, for 15 min for collagenase D or 30 min for papain. To stop the reaction, the samples were transferred at 4 °C and 10 µL of EDTA 500 mM (Invitrogen, Grand Island, NY, USA) were added. Finally, the brain tissue was dissociated mechanically with a glass pipette, filtered through a 70 μm nylon cell strainer humidified with cold DPBS and centrifuged at 300 *g* for 15 min at 4 °C. The pellet was resuspended with 25% Percoll (GE Healthcare, Uppsala, Sweden) and centrifuged at 1000 *g* for 10 min at RT to remove cell debris and myelin. The fat-containing white layer was carefully removed and the cell pellet was resuspended in the appropriate buffer for RNA sequencing (RNA-seq) or flow cytometry.

### RNA sequencing and analysis

As described previously [30], cells suspensions were incubated with FcR Blocking Reagent (1:10, Miltenyi Biotec, Bergisch Gladbach, Germany) and anti-CD11b MicroBeads (1:10, Miltenyi Biotec) in 100 µL of cytometry buffer (CB): 5 mM EDTA, 0.5% FBS (Gibco, Paisley), 100 U/ml penicillin G (Gibco), 100 µg/ml streptomycin (Gibco) in PBS for 15 min at 4 °C. CD11b^+^ cells were separated in an autoMACS Pro Separator (Miltenyi Biotec). The CD11b^−^ fraction was collected and incubated with FcR Blocking Reagent (1:10) and anti-ACSA2 MicroBeads (1:10, Miltenyi Biotec) in CB for 15 min at 4 °C and separated in an autoMACS to isolate astrocytes. CD11b^+^ and ACSA2^+^ cells were pelleted and resuspended in the lysis buffer from the Dynabeads mRNA Direct Kit (Ambion, Foster City, CA, USA). Samples were stored at -80 °C. RNA-seq was performed using MARS-seq adapted for bulk RNA-seq Include this Ref PMID24531970 and PMID28475900 with minor modifications. Poly-A RNA was extracted with Dynabeads Oligo (dT) (Thermo Fisher Scientific) and reverse transcribed with AffinityScript Multiple Temperature Reverse Transcriptase (Agilent Technologies, Santa Clara, CA, USA) using poly-dT oligos carrying a 7-base pair (bp) index. Upon indexing, samples were pooled and subjected to linear amplification using HiScribe T7 High Yield RNA Synthesis Kit (New England Biolabs, Ipswich, MA, USA). Then, the antisense RNA was fragmented into 250–350 bp fragments using RNA Fragmentation Reagents (Thermo Fisher Scientific) and dephosphorylated for 15 min at 37 °C with 1 U FastAP (Thermo Fisher Scientific). Partial Illumina adaptor sequences PMID24531970 were ligated to the fragments with T4 RNA Ligase 1 (New England Biolabs, Ipswich, MA, USA) and reverse transcription was repeated. Full Illumina adaptor sequences were added during library amplification with KAPA HiFi DNA Polymerase (Kapa Biosystems, Wilmington, MA, USA). The libraries were then quantified using a Qubit 3.0 Fluorometer (Life Technologies) and their size profiles were examined in an Agilent 4200 TapeStation System. Libraries were sequenced in an Illumina NextSeq 500 instrument at a sequence depth of 10 × 10^6^ reads/sample. The Kallisto pseudoaligner (version 0.46) was applied for *Mus musculus* transcriptome quantification. First, the fasta file of the GRCm38.p6 assembly was used to create the index file. Then, measurement of the expression was carried out with quant mode. Applied parameters were: single-end, 100 bootstrap, length = 68 and standard deviation = 20. Differentially expressed transcripts were identified through sleuth. A cut off *p*-value < 0.01 was applied to select the most relevant transcripts.

Ingenuity Pathway Analysis (IPA) software (Qiagen) was used to generate a network of biological functions, pathways and upstream regulators predicted to be altered for each differentially displayed gene set as follows. Differentially expressed transcripts generated from MPTP mouse models and human data PD downloaded from Smajić et al., 2022 [31] were imported as new “core analysis” selecting the options “flexible format” and “Ensembl” transcript identification. Next, expression *p*-value and b value of each transcript were uploaded and “expression analysis” classification (expr log ratio) was selected. The reference set was defined as the “Ingenuity Knowledge Bases genes” and the option “direct and indirect relationships” was chosen. Networks of pathways, upstream regulators and biological functions were generated using the activation Z-scores (z < 1 or z > 1) and differentially expressed genes (*p* < 0.01). For Ingenuity Comparison Analysis data obtained from IPA “expression analysis” corresponding to the midbrain were used. Murine data sets were also subjected to a gene set enrichment analysis (GSEA). The data are publicly available in NCBI’s Gene Expression Omnibus (GEO) [32] and are accessible through GEO Series accession number GSE191131.

### Flow cytometry

Cell pellets obtained from the striatum and midbrain were resuspended in 100 µL of CB and incubated for 5 min at RT with Zombie NIR Dye (BioLegend, San Diego, CA, USA) to assess cell viability (50 µL/sample; 1:2000 dilution in PBS). After quenching with CB, cells were centrifuged at 2000 rpm for 1 min, and labeled with BV510 anti-CD11b (1:500, M1/70, BioLegend), BV421 anti-CD45 (1:1000, 30F11, BioLegend), PE-Cy7 anti-CD8a (1:400, 53 − 6.7, BioLegend) or BUV395 anti-CD8a (1:200, 53 − 6.7, BD Bioscience), and FITC anti-CD4 (1:1000, GK1.5, BioLegend) or BV711 anti-CD4 (1:200, GK1.5, BioLegend) diluted in CB and the FcR Blocking Reagent (1:50; Miltenyi Biotec, Bergisch Gladbach, Germany) during 15 min at 4 °C in the dark. For intracellular staining, cells were incubated with fixation/permeabilization solution (Invitrogen) during 7 min at 4 °C in the dark and washed with PermWASH solution (Invitrogen) and incubated with FITC anti-Ki67 (1:500, 11F6, BioLegend) diluted in PermWASH solution during 15 min at 4 °C in the dark. Once labeled, samples were centrifuged, resuspended in CB and data was acquired using a CytoFLEX LX Flow Cytometer (Beckman Coulter, Brea, CA). The analysis was performed using the CytExpert 2.3 (Beckman Coulter) and the FlowJo 10.0.7r2 (BD Biosciences, Franklin Lakes, NJ) softwares.

### Histological techniques

Animals were anesthetized with i.p. ketamine/xylazine and perfused transcardially during 5 min with Ringer’s solution (145.4 mM NaCl, 3.4 mM KCl, 2.4 mM NaHCO_3_;, pH 7.4) at a rate of 9.5 mL/min and during 10 min with 4% para formaldehyde (PFA; Panreac, Barcelona, Spain) in 0.125 M PBS, pH 7.4 at the same rate, followed by 3 additional min at 16 mL/min. Brains were removed, post-fixed during 24 h in 4% PFA and stored in 30% sucrose/PBS. Coronal 40 μm thick sections were obtained using a Leica SM2000R sliding microtome (Leica, Wetzlar, Germany). Free-floating sections were washed with PBS and endogenous peroxidase activity was inactivated for 30 min with 0.03% H_2_O_2_ (Sigma-Aldrich)/methanol (Panreac). After washing the tissue with PBS, sections were incubated with the blocking solution: 4% normal goat serum and/or normal donkey serum, 0.05% Triton X-100 (Sigma Aldrich) and 4% BSA (Merck, Darmstadt, Germany) in PBS during 40 min. Then, the tissue was incubated overnight with the primary antibodies diluted in blocking solution at RT. The primary antibodies used were: rabbit anti-tyrosine hydroxylase (TH, 1:1000; Merck Millipore), sheep anti-TH (1:1000; Abcam, Cambridge), guinea pig anti-Iba1 (1:1000; Synaptic Systems, Göttingen, Germany) and mouse anti-GFAP (1:500; Cell Signaling Technology, Danvers, MA). For immunofluorescence staining, antibody binding was detected by incubating sections with the secondary antibodies diluted in blocking solution for 2 h at RT: Alexa Fluor 488 donkey anti-sheep (1:500; Jackson ImmunoResearch), Alexa Fluor 568 donkey anti-mouse (1:500; Thermo Fisher Scientific), Alexa Fluor 488 donkey anti-rabbit (1:250; Invitrogen) and Alexa Fluor 594 goat anti-guinea pig (1:500; Invitrogen, Carlsbad, CA). Finally, the tissue was stained with DAPI (1:50,000; Sigma-Aldrich). Sections were mounted on glass slides in a 0.2% solution of gelatin in 0.05 M Tris–HCl buffer (pH 7.6) (Sigma-Aldrich), dried and dehydrated in toluene (Panreac) for 12 min before coverslipping with DPX (BDH Chemicals, Poole).

### Image analysis

Confocal images of the double staining TH/Iba1 and TH/GFAP were acquired on a LSM800 confocal microscope (Zeiss, Jena, Germany) using the 20× and 63× oil objectives. A projection stack with the same number of images per slice was generated. For the morphological analysis of Iba1^+^ cells, 3 types of morphologies were considered: ramified, hypertrophic and bushy. Ramified Iba1^+^ cells present long thin processes and small cell bodies. Hypertrophic Iba1^+^ cells are characterized by long and thick processes and a big cell body. Bushy Iba1^+^ cells have a big cell body and short processes. Morphological Iba1 cells classification was made manually from four stacks obtained from each region. We generated an ImageJ plugin (Neuronphagocytosis) to calculate TH^+^, Iba1^+^ and GFAP^+^ volumes (µm^3^) defined as particles with size larger than 20 pixels. Then, we quantitated the volume of TH^+^ signal within the Iba1^+^ or GFAP^+^ volumes. The Sholl plugin was used to determine microglia ramification.

### Statistics

The normal distribution of data was analyzed with a Shapiro-Wilk test. Student’s *t*-test (two-tailed) for equal variances was used for pair-wise comparisons of data following a normal distribution. If variances were significantly different, the Welch’s correction was applied. Mann-Whitney U test was used to evaluate data that do not follow a normal distribution. One-way ANOVA was used to compare more than two groups following a normal distribution and the Kruskal-Wallis test was used for data that do not follow a normal distribution followed by Dunn’s multiple comparisons test. For multiple comparisons, the two-way ANOVA followed by Bonferroni’s post hoc test was used. Data were analyzed using GraphPad Prism version 8.0. All data are represented as mean with 95% confidence intervals (CI) and *p*-values < 0.05 were considered as statistically significant.

## Results

### The MPTP administration pattern induces different glial activation in the striatum and in the midbrain

**Fig. 1:**
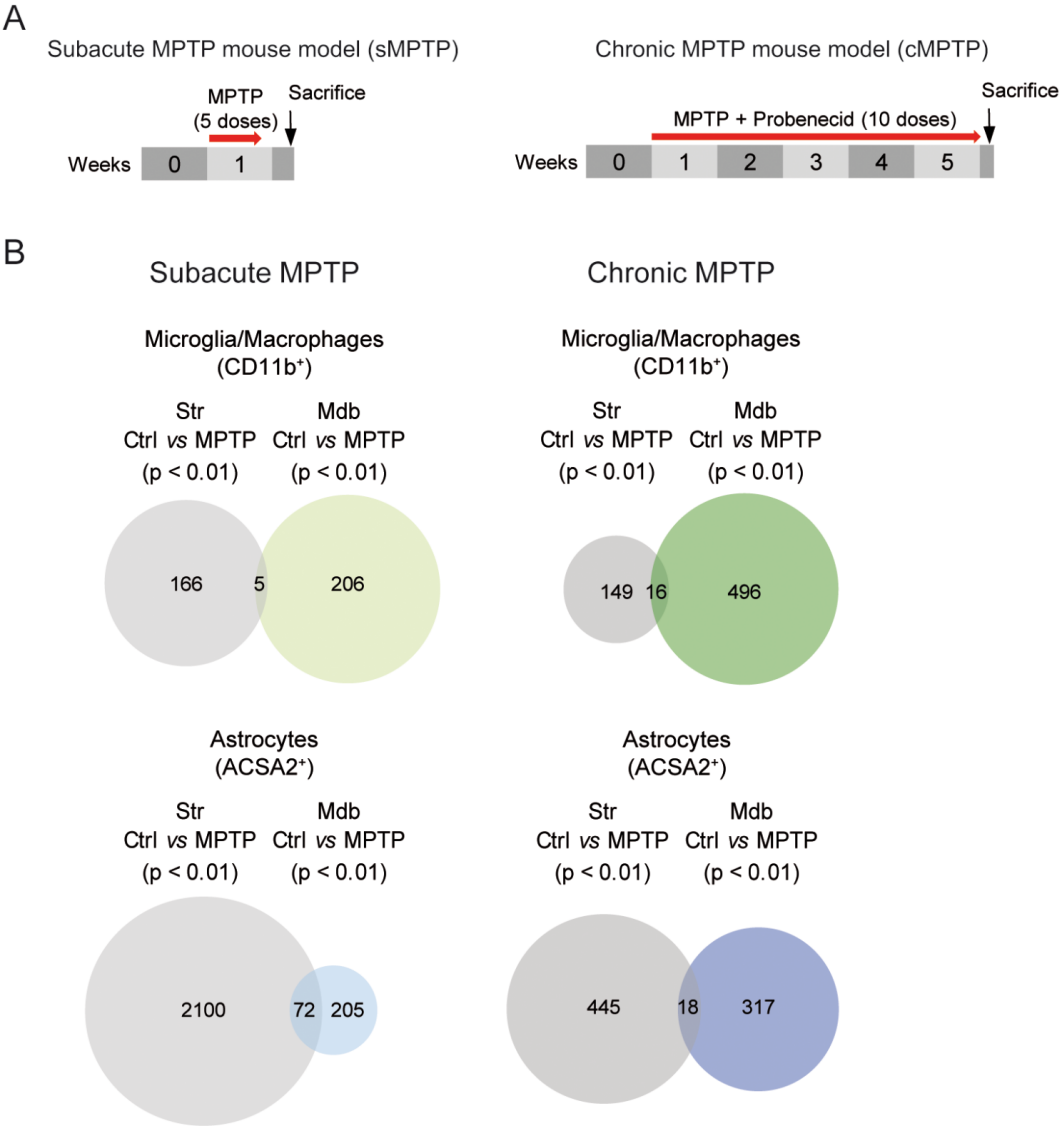
Differentially displayed transcripts in CD11b^+^ and ACSA2^+^ cells purified from the striatum and the midbrain after subacute or chronic MPTP intoxication. (**A**) Scheme of the two MPTP intoxication protocols used. In the subacute regimen (sMPTP), mice received 5 consecutive MPTP doses and in the chronic administration (cMPTP) animals received 10 MPTP doses along 5 weeks. Control mice were injected with the MPTP vehicle or MPTP vehicle with probenecid following the same MPTP administration pattern. sMPTP mice were sacrificed 24 h and cMPTP 48 h after the last MPTP injection. CD11b^+^ and ACSA2^+^ were purified from the striatum and the ventral midbrain for bulk RNA sequencing. (**B**) Venn diagrams representing differentially displayed genes (*p* < 0.01) in sMPTP and cMPTP animals with respect to their corresponding controls. (*N* = 3 animals/group). Str: striatum; Mdb: ventral midbrain

The mechanism of neuronal death induced by MPTP involves mitochondrial dysfunction and oxidative stress [33]. In this study we questioned whether the same neurotoxic stimuli, MPTP, would induce different glial activation profiles depending on the administration pattern. For this purpose, we selected two widely used MPTP administration regimens to generate experimental PD in mice. In the subacute regimen (sMPTP), 5 doses of MPTP (30 mg/kg) are injected daily for 5 consecutive days and in the chronic regimen (cMPTP), 10 doses of the neurotoxin (20 mg/kg) are co-administered with probenecid to prevent renal clearance, twice a week along 5 weeks (Fig. 1A). The different administration patterns would induce dopaminergic neuron degeneration at different rates. Upon sacrifice of the animals, the striatum and the ventral midbrain were dissected out to prepare a cell suspension from each region. CD11b^+^ cells and ACSA2^+^ astrocytes were subsequently separated for bulk mRNA sequencing. Due to MPTP-induced degeneration, glial cells presented different activation profiles in each region that varied with the MPTP intoxication regimen (Fig. 1B). CD11b^+^ cells showed increased differentially displayed transcripts in the midbrain compared to the striatum and these differences were exacerbated with the chronicity of MPTP administration (Fig. 1B). By contrast, ACSA2^+^ astrocytes showed profound rearrangements in gene expression in the striatum that decreased with the chronicity of MPTP administration (Fig. 1B). The low number of commonly triggered genes upon damage for each cell type indicates that glial cells react differently to the type or the intensity of damage.

Next, we questioned whether the transcriptomic profile of glial cells in the context of nigrostriatal damage presented common features with other pathological conditions. Different signatures were selected for a gene set enrichment analysis (GSEA). The activation state of CD11b^+^ cells was compared with the neurodegenerative microglia (MGnD, ) which was established from different mouse models of neurological diseases including ALS, AD and multiple sclerosis (MS) [34]; with disease-associated microglia (DAM) described in an AD and ALS transgenic mouse models [35]; and with a specific subset of phagocytic microglia described in the same AD model [36](Table 1). The differentially displayed genes in CD11b^+^ cells from parkinsonian animals failed to show a MGnD profile except for the downregulated transcripts in the midbrain of sMPTP mice. DAM microglia were identified exclusively in striatal CD11b^+^ cells from cMPTP mice. Striatal and midbrain CD11b^+^ cells from sMPTP mice showed downregulation of genes that are also reduced in DAM, which may be attributed to the decreased expression of homeostatic microglial genes associated with neuronal loss [37] (Table 1). Phagocytic CD11b^+^ cells similar to that described in AD models were identified in the midbrain of cMPTP mice. In the striatum, CD11b^+^ cells shared upregulated, but not downregulated, transcripts with the microglial phagocytic phenotype (Table 1). The absence of a phagocytic profile in CD11b^+^ cells in the midbrain of sMPTP mice would reflect different activation patterns in response to the type of neuronal death [33, 38]. To delve into the phagocytic phenotype, we used an additional gene set obtained from gene ontology (GOBP_Phagocytosis, GO0006909, MM4904). Only striatal CD11b^+^ cells of cMPTP mice shared transcripts with this signature, the low correlation with the rest of CD11b^+^ cell subsets suggests that specific phagocytic processes are triggered by neurodegeneration or that these cells are at different stages of the process.

**Table 1:** *p* values corresponding to the GSEA analysis performed to compare striatal and midbrain differentially displayed genes in CD11b^+^ cells with signatures that represent different microglial states

		sMPTP		cMPTP	
Signatures		Striatum	Midbrain	Striatum	Midbrain
MGnD	Up	0.945	0.625	0.423	0.127
	Down	0.476	0.036*	0.234	0.226
DAM	Up	0.093	0.985	0.005**	0.057
	Down	< 0.001***	< 0.001***	0.004**	< 0.001***
Phagocytic microglia	Up	0.001**	0.746	< 0.001***	< 0.001***
	Down	0.051	0.279	0.252	0.028*
Phagocytosis (GOBP)		0.494	0.836	0.029*	0.075

The activation profile of ACSA2^+^ cells obtained from parkinsonian animals was compared to an LPS-induced neurotoxic phenotype and to a beneficial phenotype identified in ischemia [39]. Midbrain astrocytes in sMPTP mice presented the neurotoxic phenotype not detected in the striatum or in cMPTP mice. By contrast, the expression profile of striatal astrocytes correlated strongly with the GOBP phagocytosis gene set (Table 2).

**Table 2:** *p* values corresponding to the GSEA analysis performed to compare striatal and midbrain differentially displayed genes in ACSA2^+^ cells with signatures that represent different astroglial states

	sMPTP		cMPTP	
	Striatum	Midbrain	Striatum	Midbrain
LPS reactive (A1)	0.521	0.029*	0.248	0.467
Ischemic (A2)	0.731	0.887	0.182	0.923
Phagocytosis (GOBP)	< 0.001***	0.535	0.006**	0.054

Selection of genes from the phagocytic microglia and the GOBP Phagocytic gene sets expressed in CD11b^+^ and ACSA2^+^ cells allowed us to elaborate a heatmap that confirms the phagocytic profile except for CD11b^+^ cells in sMPTP mice (Suppl. Figure 1). These results suggest that degeneration of the nigrostriatal pathway polarizes ACSA2^+^ cell activity towards a neuroinflammatory and phagocytic phenotype. CD11b^+^ cells are involved in the removal of striatal terminals and neuronal cell bodies in the cMPTP.

**Fig. 2:**
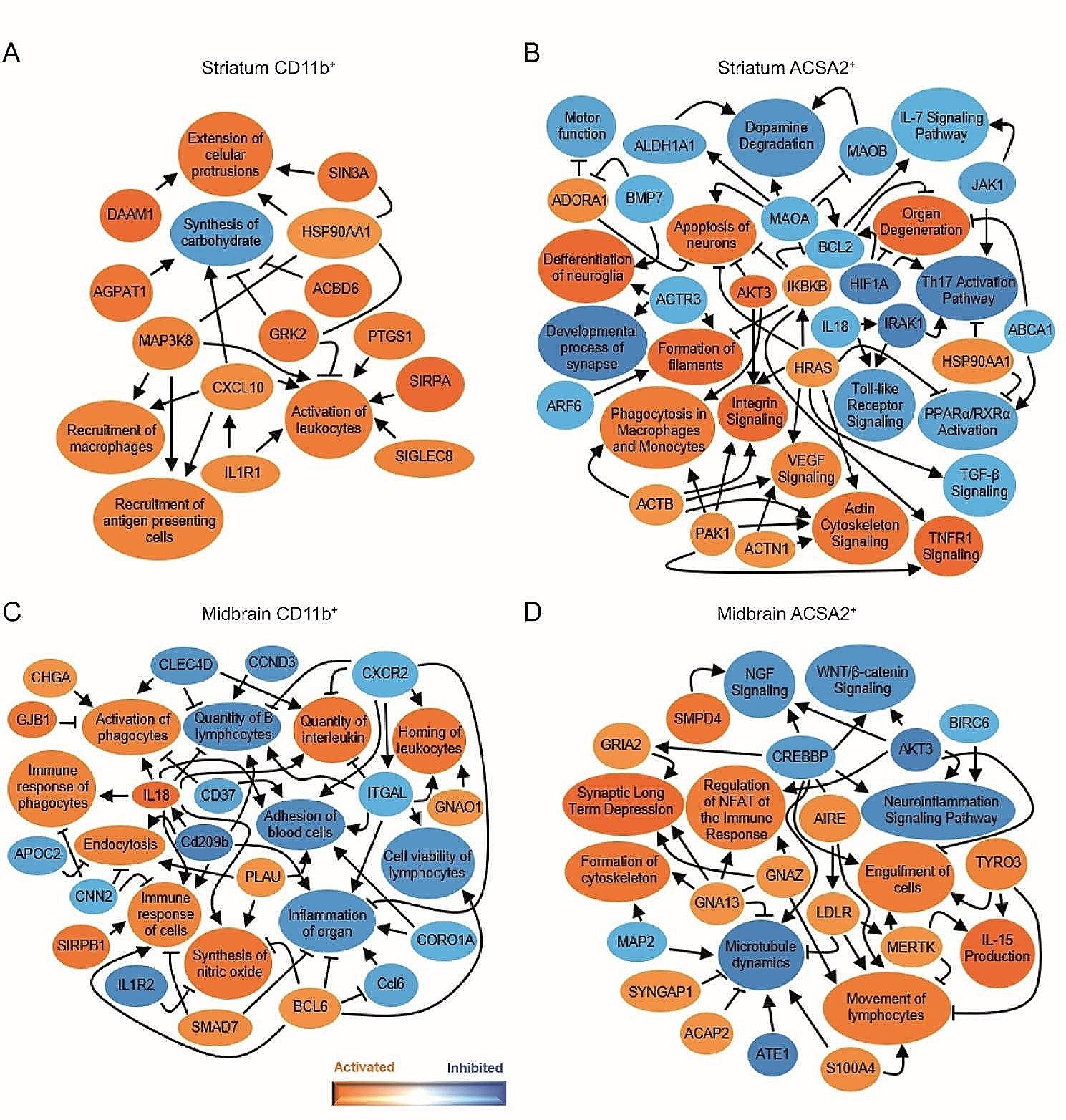
Graphical summary of networks predicted to be altered in glial cells of the sMPTP mice. Ingenuity pathway analysis yielded networks of pathways, upstream regulators and biological functions predicted to be altered (z < 1 or z > 1) and differentially expressed genes (*p* < 0.01) in: (**A**) striatal CD11b^+^, (**B**) striatal ACSA2^+^, (**C**) midbrain CD11b^+^ and (**D**) midbrain ACSA2^+^ cells

To gain further insight into the activation state of glial cells under parkinsonian conditions, we generated a network of biological functions, pathways and upstream regulators predicted to be altered for each differentially displayed gene set with Ingenuity Pathway Analysis (IPA). The network obtained for the striatum of sMPTP mice showed that most of the predicted pathways activated in CD11b^+^ cells are related to phagocytosis/removal of pathogens (*Daam1*, *Agpat1, Siglec8*), and to the recruitment of immune cells/production of pro-inflammatory signals (*Ilr1, Cxcl10, HSP90AA1, Map3k8, Grk2, Ptgs1*) (Fig. 2A). ACSA2^+^ cells in this region are also involved in inflammation, the downregulation of pro-inflammatory pathways such as IL7, IL17, IL18 or toll like receptor (TLR), together with the activation of TNFR1 and inhibition of TGFβ signaling, suggest that these cells are playing a relevant role in containing the pro-inflammatory reaction (Fig. 2B). Additional activated pathways revealed that astrocytes are experiencing profound morphological changes (*Actb, Actn1, Pak1*) and are adapting to changes in dopamine metabolism (*MaoA, MaoB, Adora1*) (Fig. 2B). In the midbrain, the predicted decrease in *Ilr2, Cxcr2, Ccl6, Cd209b, Itgal, Clec4D, Ccnd3, Coro1A* and the increase in *Bcl6* signaling in CD11b^+^ cells suggests that they are involved in the downregulation of pro-inflammatory signals and adaptive immune cell infiltration (Fig. 2C). In the same context, ACSA2^+^ cells undergo a reprogramming process that affects astrocyte-neuron interactions (*Gria2, Map2, Syngap1, Acap2*), activates a phagocytic program (*Mertk, Tyro3*), promotes pro-inflammatory responses (*S100A4*) and protective inflammatory pathways mediated by IL15 and regulated by *Aire*. Concomitantly, classical neuroprotective signaling pathways such as the nerve growth factor and the WNT/β-catenin are downregulated (Fig. 2D).

**Fig. 3:**
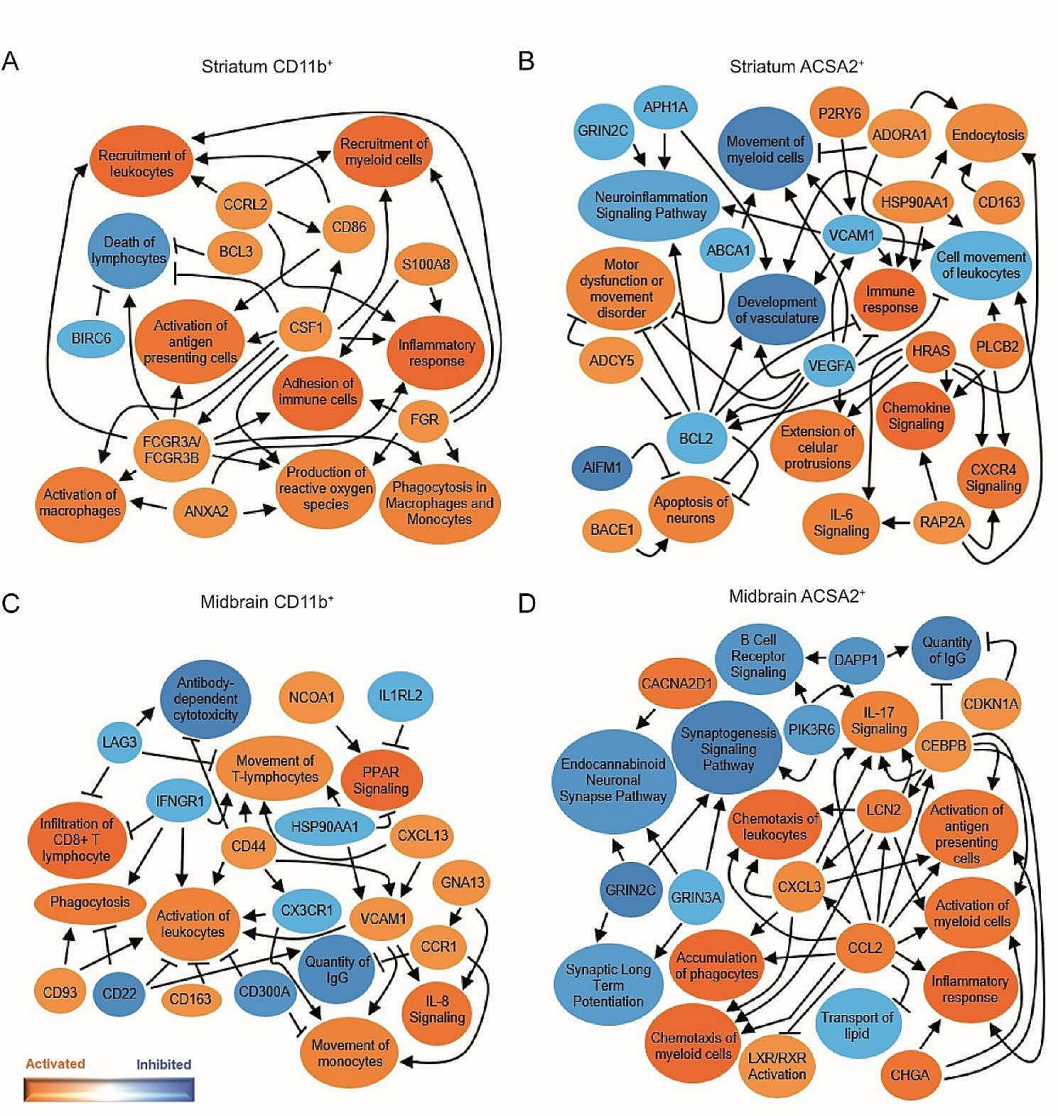
Graphical summary of networks predicted to be altered in glial cells of the cMPTP mice. Ingenuity pathway analysis yielded networks of pathways, upstream regulators and biological functions predicted to be altered (z < 1 or z > 1) and differentially expressed genes (*p* < 0.01) in: (**A**) striatal CD11b^+^, (**B**) striatal ACSA2^+^, (**C**) midbrain CD11b^+^ and (**D**) midbrain ACSA2^+^ cells

An equivalent analysis carried out in cMPTP mice revealed that, although the mediators are different, striatal CD11b^+^ cells also exhibit a phagocytic (increased *Fgcr3*, decreased *Birc6* signaling) and pro-inflammatory (increased *Ccrl2, Cd86, Fgr, S100a8*) functionality (Fig. 3A). ACSA2^+^ cells expressed mainly pathways related to astrocyte/neuron communication (*Grinc2c, Adcy5, Aph1a, Aifm1, Bace1, Adora1*) and an increase of IL6 and CXCR4 signaling pathways (Fig. 3B). In the midbrain, immune modulating pathways that involve *Cd300a, Cd93, Cd22* or *Lag3* together with increased *Cd44, Cxcl13, Ccr1, Hso90aa1, Vcam1, Gna13* and decreased *Infgr1* or *Ilr1l2* pro-inflammatory signals are altered in CD11b^+^ cells. Overall, the predicted signaling suggests that these cells are contributing to the inflammatory reaction and immune cell infiltration in this region (Fig. 3C). In the same context, ACSA2^+^ cells decreased pathways involved in synaptic plasticity and activated a pro-inflammatory profile through the increase in IL17 signaling and *Ccl2, Il17, Chga, Cebpb, Dapp1* (Fig. 3D). Altogether, these results suggest that in the striatum CD11b^+^ cells are the main drivers of the pro-inflammatory reaction, independently of the MPTP intoxication pattern. In the midbrain of sMPTP mice, CD11b^+^ cells show an anti-inflammatory response that turns into pro-inflammatory upon damage chronification. Through different mediators, ACSA2^+^ cells exhibit a loss of homeostatic functions and a pro-inflammatory profile in the midbrain of sMPTP and cMPTP mice.

### Involvement of glial cells in the phagocytosis of dopaminergic cell debris

**Fig. 4:**
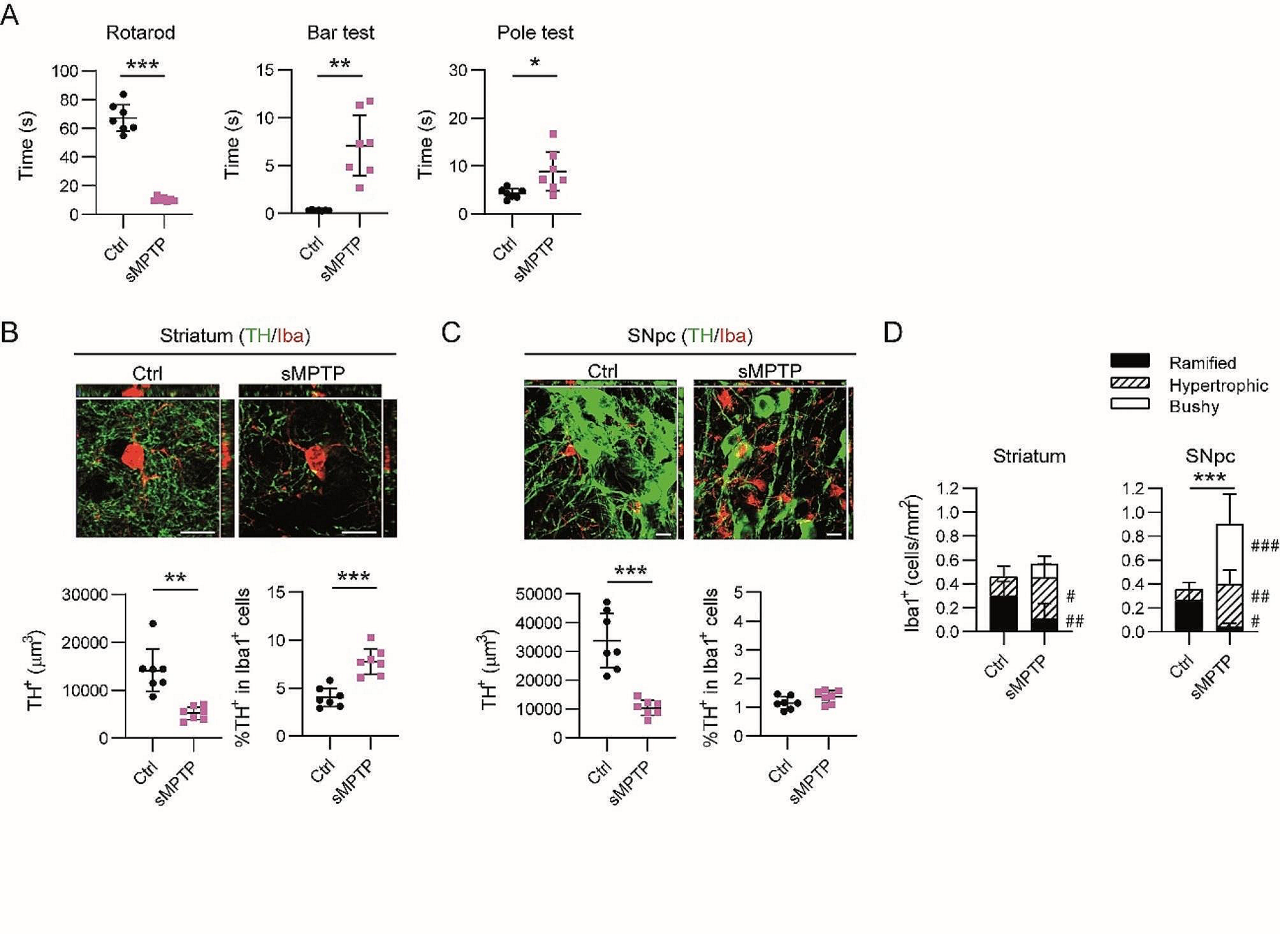
Phagocytic assessment of Iba1^+^ cells in sMPTP mice. Animals were sacrificed at 10 days after the first MPTP injection. (**A**) Motor behavior was evaluated in the rotarod, bar and pole test. (**B** and **C**) Representative images of a single plane and the corresponding orthogonal projection of the stack of TH/Iba1 double immunofluorescence in (**B**) the striatum and (**C**) the SNpc of control and sMPTP mice. Quantitation of TH^+^ volume and percentage of TH^+^ signal in Iba1^+^ cells. (**D**) Quantitation of the number of ramified, hypertrophic and bushy Iba1^+^ cells in the striatum and SNpc from control and sMPTP mice. Data represent the mean ± 95% CI from 7 animals per group. Statistical analysis: (**A**, **B** and **C**) t-test with Welch correction when variances differed significantly, (**D**) 2-way ANOVA followed by Bonferroni post hoc test. Statistical significance in, (*) cell number, (^#^) cell morphology. *^/#^*p* < 0.05, **^/##^*p* < 0.01, ***^/###^*p* < 0.001. Magnification bars: 10 μm

To delve into the differential phagocytic profile of CD11b^+^ cells in the SNpc, we prepared a new set of animals which was processed for histological techniques. Mice receiving the sMPTP experienced motor impairment measured in the rotarod, bar test and pole test (Fig. 4A). A decrease in TH^+^ terminals that could reflect the loss of dopaminergic terminals was detected in the striatum of sMPTP animals (Fig. 4B). Phagocytosis, quantitated as the fraction of TH^+^ signal measured inside Iba1^+^ cells, increased significantly in this region (Fig. 4B). In the SNpc, the significant decrease in TH immunostaining was not accompanied by an increase in the phagocytic activity (Fig. 4C). This data correlate with the absence of a phagocytic transcriptomic profile in CD11b^+^ cells (Table 1). The increased number and morphological changes of Iba1^+^ cells in the SNpc (Fig. 4D) indicate that these cells are reacting to dopaminergic damage. Iba1^+^ were classified based on their shape as ramified, hypertrophic and bushy (Suppl. Figure 2). Control animals presented a predominant subset of ramified Iba1^+^ cells and a minor population of hypertrophic cells. Upon sMPTP administration, Iba1^+^ cells polarized towards a hypertrophic phenotype accompanied by a decrease of the ramified morphology and a new population of bushy cells was detected mainly in the SNpc (Fig. 4D).

**Fig. 5:**
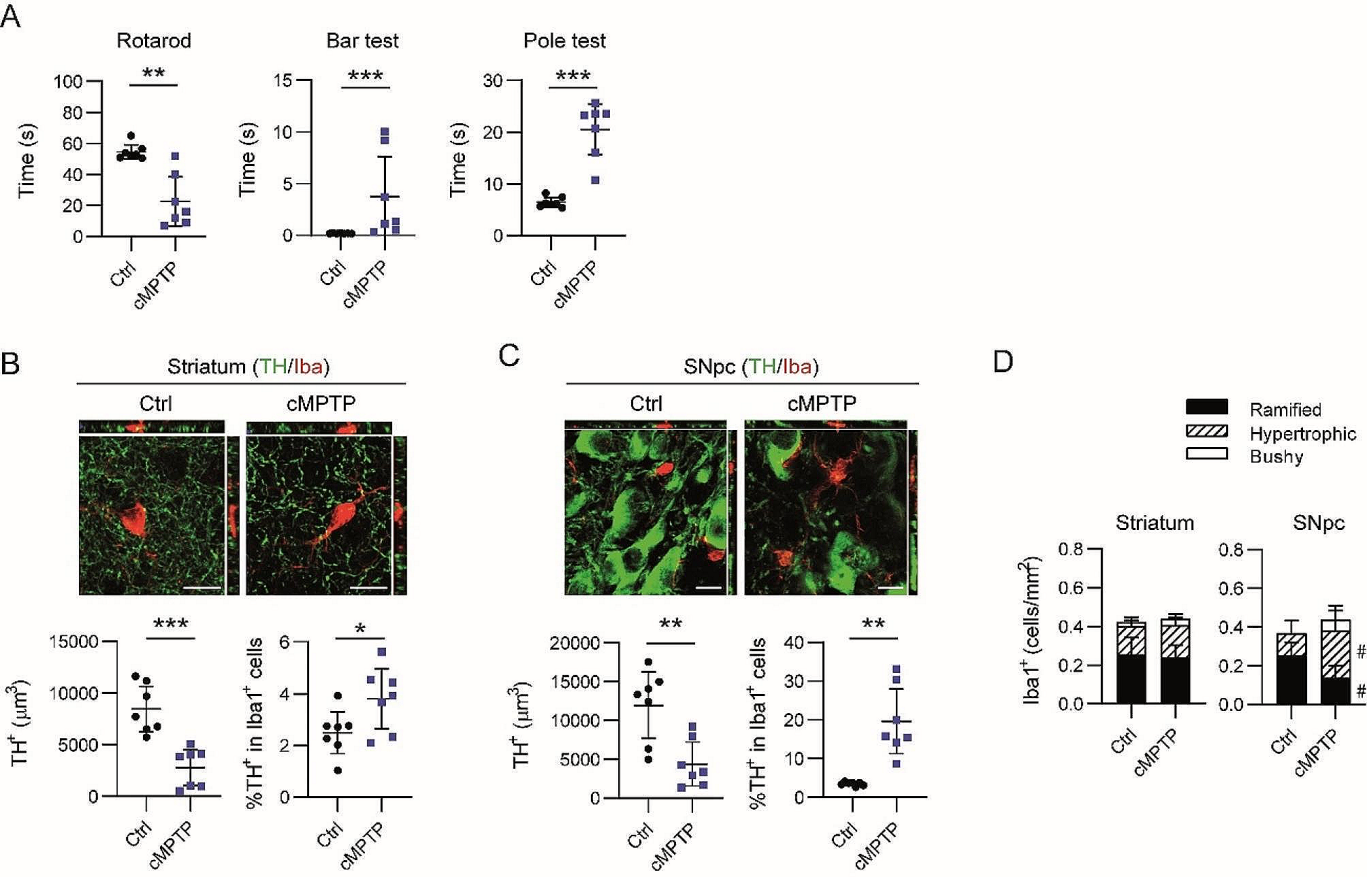
Phagocytic assessment of Iba1^+^ cells in cMPTP mice. Animals were sacrificed at 5 weeks after the first MPTP injection. (**A**) Motor behavior was evaluated in the rotarod, bar and pole test. (**B** and **C**) Representative images of a single plane and the corresponding orthogonal projection of the stack of TH/Iba1 double immunofluorescence in (**B**) the striatum and (**C**) the SNpc of control and cMPTP mice. Quantitation of TH^+^ volume and percentage of TH^+^ signal in Iba1^+^ cells. (**D**) Quantitation of the number of ramified, hypertrophic and bushy Iba1^+^ cells in the striatum and SNpc from control and cMPTP mice. Data represent the mean ± 95% CI from 7 animals per group. Statistical analysis: (**A**, **B** and **C**) t-test with Welch correction when variances differed significantly, (**D**) 2-way ANOVA followed by Bonferroni post hoc test. Statistical significance in (^#^) cell morphology. ^#^*p* < 0.05. Magnification bars: 10 μm

Mice receiving the cMPTP regimen exhibited motor impairment in the rotarod, bar test and pole test (Fig. 5A), and a decreased TH immunostaining in the striatum (Fig. 5B) and in the SNpc (Fig. 5C) corresponding to a damaged nigrostriatal pathway. Phagocytic activity of Iba1^+^ cells increased in the striatum (Fig. 5B) and in the SNpc (Fig. 5C). At this stage, the number of Iba1^+^ cells remained constant in both regions, independently of the parkinsonian conditions, and no morphological changes were appreciated in the striatum (Fig. 5D). By contrast, an increased fraction of Iba1^+^ cells in the SNpc showed a hypertrophic and bushy morphology together with a reduction of the ramified phenotype (Fig. 5D). These results suggest that phagocytosis of neuronal cell bodies in the SNpc is a late event.

**Fig. 6:**
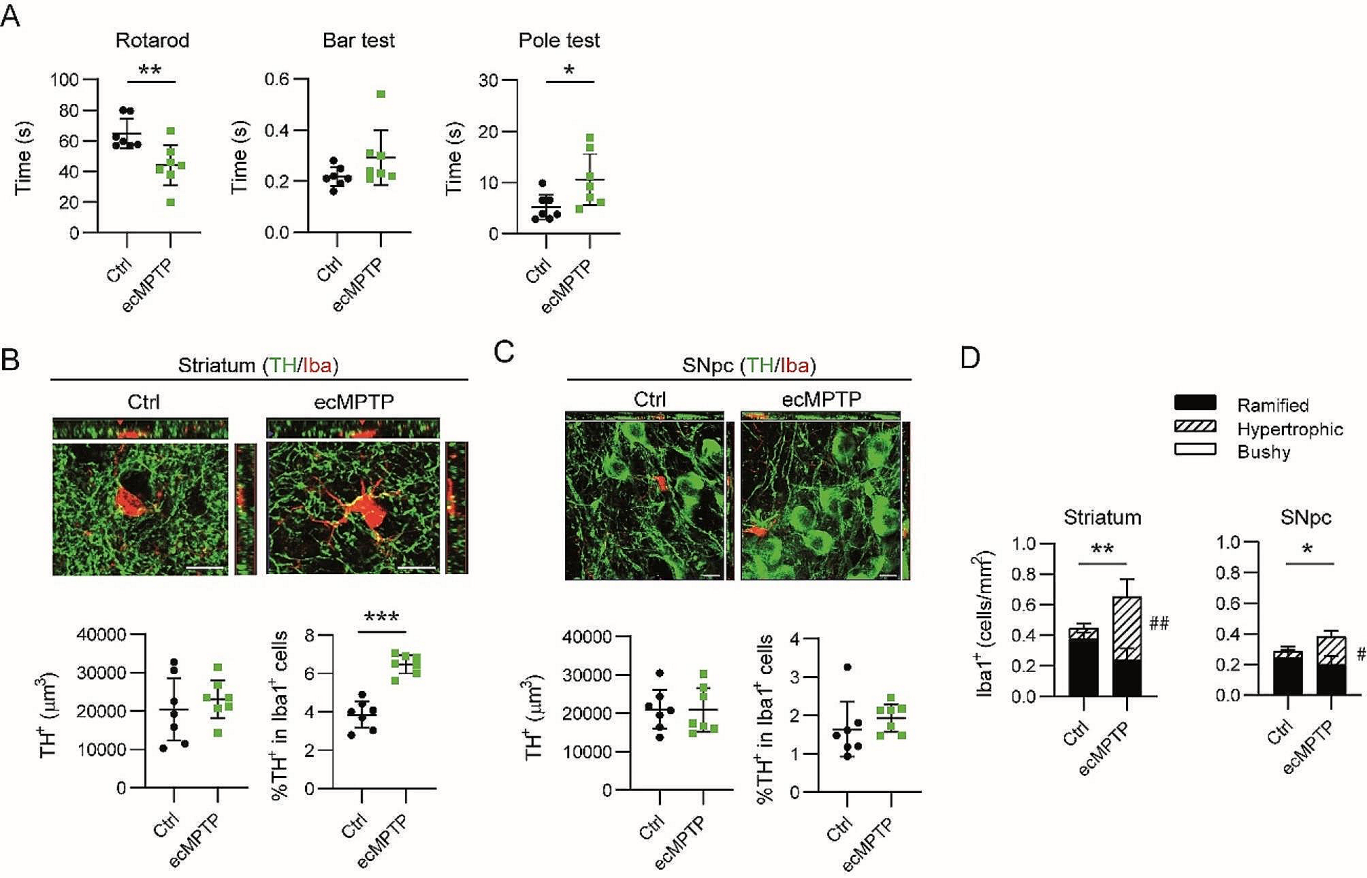
Phagocytic assessment of Iba1^+^ cells in ecMPTP mice. Animals were sacrificed at 10 days after the first MPTP injection following the cMPTP intoxication pattern. (**A**) Motor behavior was evaluated in the rotarod, bar and pole test. (**B** and **C**) Representative images of a single plane and the corresponding orthogonal projection of the stack of TH/Iba1 double immunofluorescence in (**B**) the striatum and (**C**) the SNpc of control and ecMPTP mice. Quantitation of TH^+^ volume and percentage of TH^+^ signal in Iba1^+^ cells. (**D**) Quantitation of the number of ramified, hypertrophic and bushy Iba1^+^ cells in the striatum and SNpc from control and ecMPTP mice. Data represent the mean ± 95% CI from 7 animals per group. Statistical analysis: (**A**) Mann-Whitney test for the rotarod and bar test, t-test for the pole test; (**B** and **C**) t-test, (**D**) 2-way ANOVA followed by Bonferroni post hoc test. Statistical significance in, (*) cell number, (^#^) cell morphology. *^/#^*p* < 0.05, **^/##^*p* < 0.01. Magnification bars: 10 μm

Next, we questioned whether the different phagocytic activity of Iba1^+^ cells in the SNpc was determined by the MPTP administration pattern or by the time of sacrifice. Thus, we prepared a new set of animals following the cMPTP regimen that was sacrificed 10 days after the first MPTP injection (3 MPTP/probenecid doses). We termed this group “early chronic MPTP” (ecMPTP). At this point, mice experienced a mild motor impairment that was detectable in the rotarod and in the pole test (Fig. 6A). The lack of differences in TH immunostaining in the striatum (Fig. 6B) and in the SNpc (Fig. 6C) indicated that the nigrostriatal pathway was still preserved. Phagocytic Iba1^+^ cells were detected in the striatum (Fig. 6B), but not in the SNpc (Fig. 6C), corroborating the results obtained in the sMPTP suggesting that Iba1^+^ cells do not activate an early phagocytic program in the SNpc after MPTP damage. The increased number of cells in both regions and the polarization of Iba1^+^ cell morphology towards a hypertrophic phenotype (Fig. 6D) indicate that these cells sense alterations in the nigrostriatal pathway even in the absence of a noticeable decrease in TH expression. An additional Sholl analysis performed to determine Iba1^+^ cell ramification showed that in the striatum, microglia from cMPTP mice presented the highest degree of ramification (Suppl. Figure 3A). By contrast, in the SNpc the highest extension of microglia branches was detected in the ecMPTP condition (Suppl. Figure 3B). These results agree with the appearance of a new bushy phenotype with a low level of ramifications. Altogether, these results indicate that morphological changes in microglia reflect a change in the basal activation state, but do not correlate with the phagocytic activity.

**Fig. 7:**
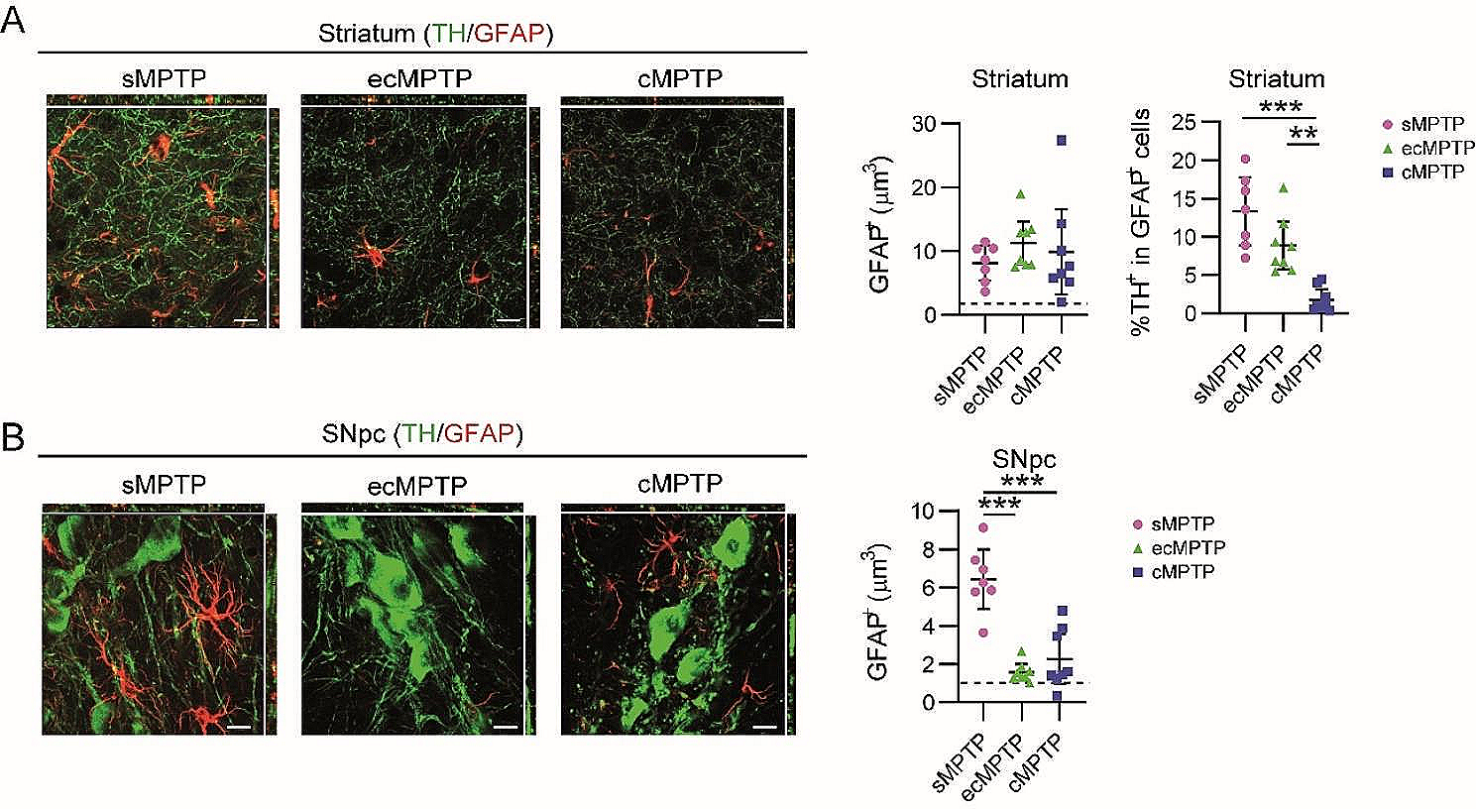
Phagocytic assessment of astrocytes in MPTP mice. Animals were sacrificed at 10 days (sMPTP and ecMPTP) or at 5 weeks (cMPTP) after the first MPTP injection. (**A**) Representative images of a single plane and the corresponding orthogonal projection of the stack of TH/GFAP double immunofluorescence in the striatum of sMPTP, ecMPTP and cMPTP mice. Quantitation of GFAP^+^ volume and percentage of TH^+^ signal in GFAP^+^ cells. Dashed line corresponds to the mean of GFAP^+^ volume in control animals. (**B**) Representative images of a single plane and the corresponding orthogonal projection of the stack of TH/GFAP double immunofluorescence in the SNpc of sMPTP, ecMPTP and cMPTP mice. Quantitation of GFAP^+^ volume. Dashed line corresponds to the mean of GFAP^+^ volume in control animals. Data represent the mean ± 95% CI from 7–8 animals per group. Statistical analysis: (**A**) One-way ANOVA test. **p < 0.01, ***p < 0.001. Magnification bars: 10 μm

Finally, the phagocytic activity of astrocytes was studied under the different MPTP administration/time regimens. Since striatal GFAP immunoreactivity is almost absent in control animals, only parkinsonian mice were selected for this analysis. Astrogliosis, measured as the volume of GFAP^+^ signal, was similarly observed in the striatum of the different models of parkinsonism (Fig. 7A). An active phagocytic process was detected at early stages of dopaminergic terminal impairment, in the sMPTP and ecMPTP mice, but not when damage was established (cMPTP) (Fig. 7A). The lack of GFAP immunoreactivity in the SNpc of ecMPTP and cMPTP mice (Fig. 7B) did not allow to carry out a similar analysis in this region. In summary, our data show that both, Iba1^+^ and GFAP^+^ cells, participate in the removal of impaired striatal terminals at the onset of the neurodegenerative process (sMPTP and ecMPTP). As the process chronifies, only Iba1^+^ cells maintain their phagocytic activity. In the SNpc the scenario is different, a lack of phagocytic microglia is observed in the sMPTP (in agreement with the GSEA analysis) and ecMPTP mice. The transcriptomic profile of ACSA2^+^ cells shows an increased expression of the phagocytic receptor *Mertk* suggesting that astrocytes may initiate the process of removal of damaged cells in this region. Polarization of microglial activity towards a phagocytic phenotype is a late event in the SNpc.

### Immune cell infiltration in the striatum and in the ventral midbrain

**Fig. 8:**
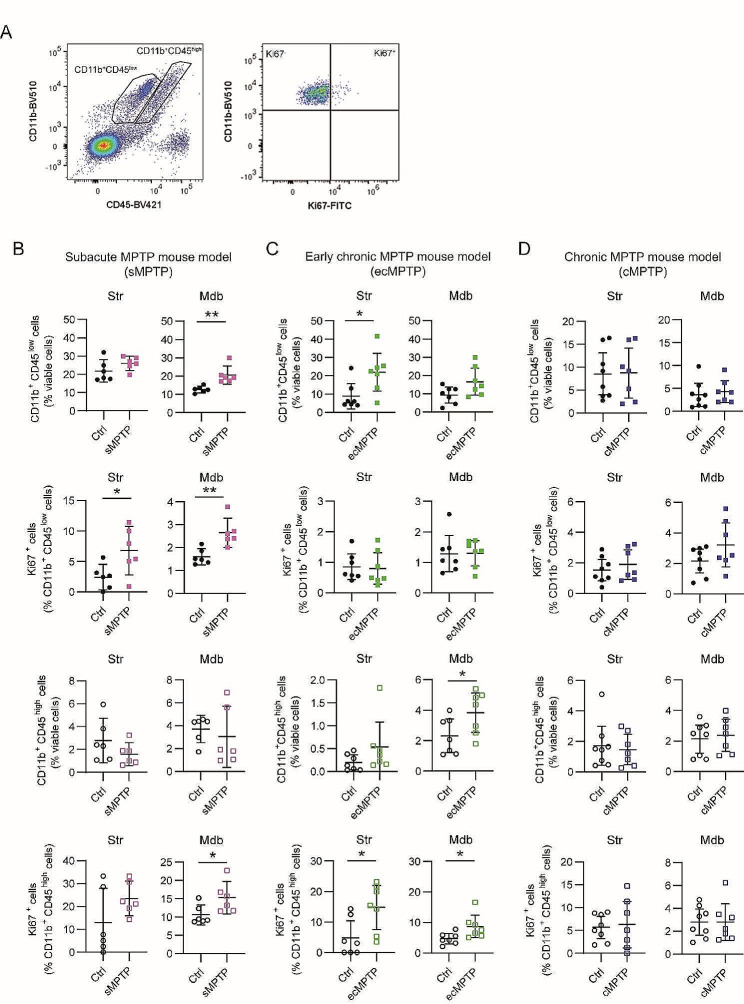
Flow cytometry analysis of CD11b^+^ cells from the striatum and in the ventral midbrain after MPTP intoxication. (**A**) Gating strategy for CD11b^+^ cell subsets differentiated based on the level of CD45 expression, CD11b^+^CD45^low^ and CD11b^+^CD45^high^, and Ki67 expression. The fraction of CD11b^+^CD45^low^ and CD11b^+^CD45^high^ cells out of viable cells and frequency of Ki67^+^ cells in these populations were analyzed in the striatum and midbrain of (**B**) sMPTP, (**C**) ecMPTP and (**D**) cMPTP mice. Data represent the mean ± 95% CI from 6 to 8 animals/group. Statistical analysis: (**B**-**D**) t-test for data following a normal distribution and Mann-Whitney test for data not following a normal distribution. *p < 0.05, **p < 0.01

Next, we questioned whether the inflammatory profile of CD11b^+^ cells in the striatum and ventral midbrain could be related to immune cell infiltration. In parallel we investigated the source of the increased number of Iba1^+^ cells. Thus, we prepared cell suspensions from the striatum and the ventral midbrain that were analyzed by flow cytometry. CD11b^+^ cells were classified based on the cell surface expression of CD11b and CD45, the CD11b^+^CD45^low^ gate was ascribed to resident microglia and the CD11b^+^CD45^high^ to infiltrated myeloid cells and resident microglia (Fig. 8A) that, under pathological conditions, are known to upregulate CD45 expression [40, 41]. In line with the Iba1^+^ cell counts, the proportion of CD11b^+^CD45^low^, but not CD11b^+^CD45^high^, was significantly increased exclusively in the midbrain of sMPTP animals. The significant difference in Ki67^+^ cells in this region suggests that cell proliferation, rather than monocyte/macrophage infiltration from the periphery, may account for this increase (Fig. 8B). The increase in Ki67^+^ observed in striatal CD11b^+^CD45^low^ and in CD11b^+^CD45^high^ midbrain cells, that was not accompanied by an alteration in cell subsets (Fig. 8B), may indicate an accelerated turnover. However, a significant increase in the fraction CD11b^+^CD45^low^ in the striatum and CD11b^+^CD45^high^ in the midbrain of ecMPTP mice could be due to cell proliferation of CD11b^+^CD45^high^ subset (Fig. 8C). The subpopulations of CD11b^+^ and Ki67^+^ cells remained constant in cMPTP animals (Fig. 8D). These results validate our histological observation of an expansion in the number of Iba1^+^ cells in SNpc in the early stages of the degeneration, which could be explained by the proliferation of CD11b^+^CD45^low^ or CD11b^+^CD45^high^ cells.

**Fig. 9:**
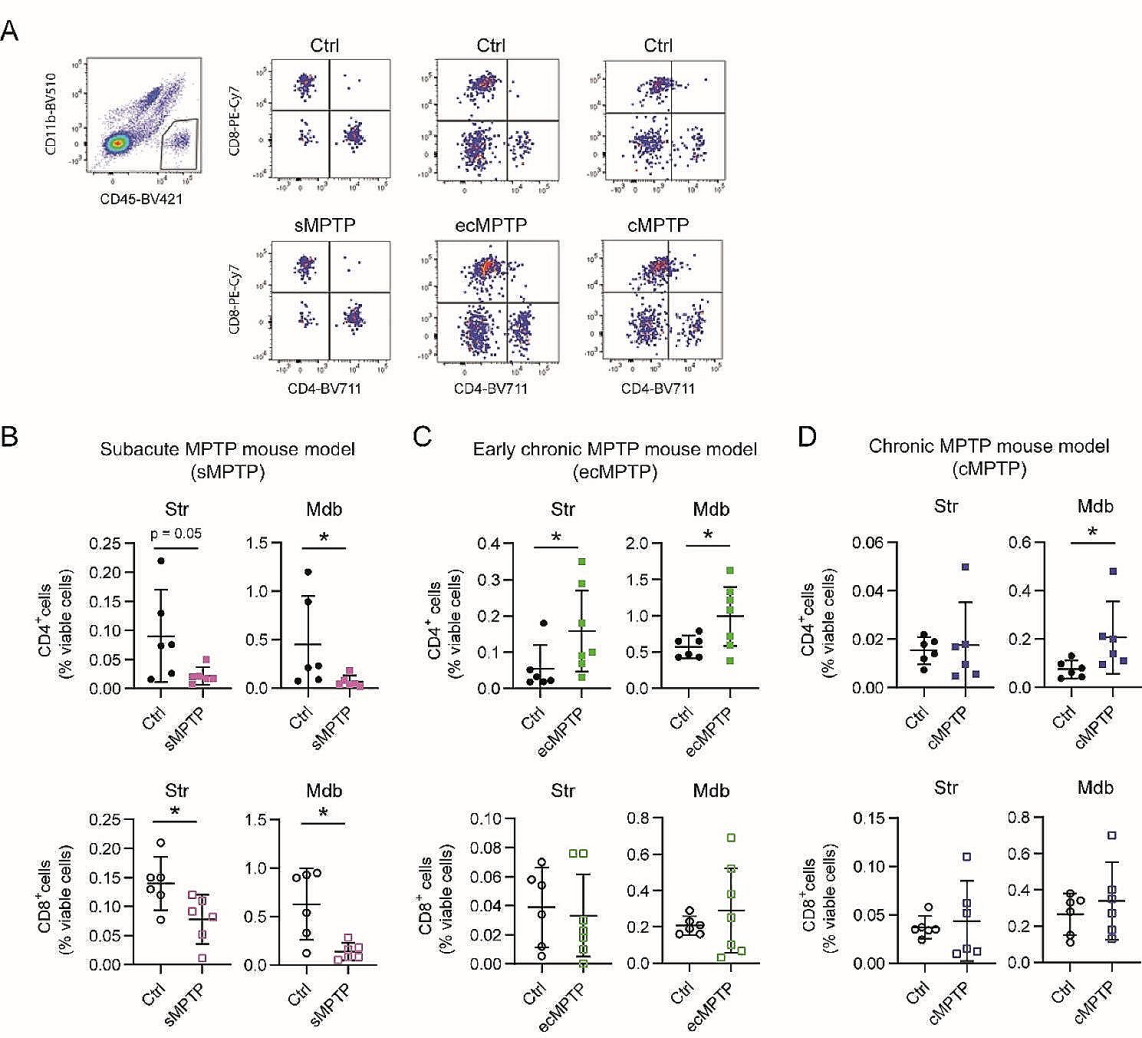
Flow cytometry analysis of lymphocyte infiltration in the striatum and in the ventral midbrain after MPTP intoxication. (**A**) Representative gating strategy to select CD11b^−^CD45^high^ population, and then CD4^+^ and CD8^+^ cells in cells suspension prepared from the ventral midbrain. Fraction of CD4^+^ and CD8^+^ cells out of viable cells in the striatum and midbrain of (**B**) sMPTP, (**C**) ecMPTP and (**D**) cMPTP mice. Data represent the mean ± 95% CI from 6 animals/group. Statistical analysis: (B-D) t-test for data following a normal distribution with Welch correction when variances differed significantly. Mann-Whitney test for data not following a normal distribution. *p < 0.05

Many of the biological functions predicted in the IPA analysis to be activated in glial cells were related to immune system infiltration and inflammation. To determine the output of these inflammatory signals, we examined T-cell infiltration within each region. The gating strategy is illustrated in Fig. 9A. Predicted pathways in striatal ACSA2^+^ cells and in midbrain CD11b^+^ cells of sMPTP mice pointed towards a downregulation of pro-inflammatory signals that could be responsible for the decreased infiltration of CD4 and CD8 T cells at this stage of degeneration (Fig. 9B). An increased CD4 T cell infiltration was present in the ecMPTP mice (Fig. 9C). The pro-inflammatory pathways activated in the midbrain CD11b^+^ cells after cMPTP administration may underly the significant increased CD4 T cell infiltration observed in this region (Fig. 9D), suggesting that lymphocyte infiltration is an early event under mild degenerative conditions. Altogether, these results indicate that the inflammatory signals generated in the context of MPTP intoxication correlate with the intensity of neuronal injury. Acute and intense dopaminergic impairment in the sMPTP mice would generate an anti-inflammatory response, while mild and chronic damage observed in cMPTP animals would induce a pro-inflammatory response detectable even at the early stages of the neurodegenerative process.

### Activation state of microglia and astrocytes in the human brain with Parkinson´s disease

**Fig. 10:**
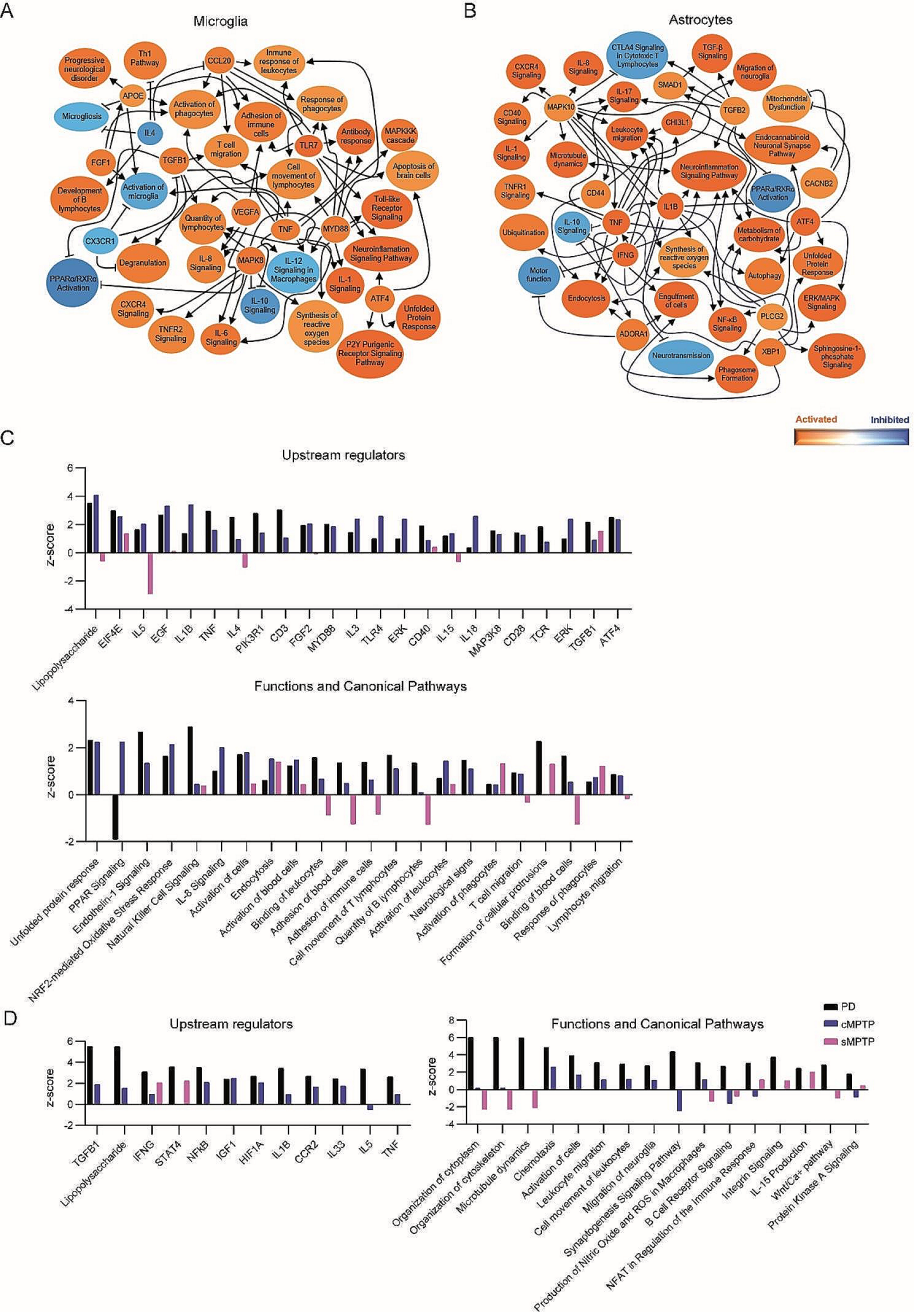
Summary of the networks predicted to be altered in human microglia and astrocytes in the midbrain. Differentially displayed genes in human glia obtained from the midbrain of PD patients were subjected to Ingenuity Pathway Analysis (IPA). The graphical summary represents the pathways, upstream regulators and biological functions predicted to be altered (**A**) in microglia and (**B**) astrocytes. The results of IPA analyses of human, sMPTP and cMPTP glial cells in the midbrain were subjected to Ingenuity Comparison Analysis to compare the z-scores of upstream regulators, pathways and biological functions in (**C**) microglia and (**D**) astrocytes

The subacute and chronic MPTP administration protocols induce different glial reactivity, inflammatory profile and immune cell infiltration into the midbrain parenchyma. To understand which activation pattern reflects better the state of these cells in PD, we took advantage of single nuclei RNA sequencing (snRNA-seq) published dataset obtained from the midbrain of 6 idiopathic PD patients with severe neuronal loss and 5 aged-matched control subjects [31]. Differentially displayed transcripts from microglia and astrocytes were analyzed as data obtained from MPTP mouse models. The IPA network for the differentially displayed genes expressed by cells annotated as microglia (*CD74*) and astrocytes (*AQP4*) was generated following the same criteria used for the MPTP pre-clinical models. Predicted activated pathways in microglia showed a clear pro-inflammatory profile related to Th1 immune pathways and TNF, IL1, IL6, IL8, CCL20, TLR or CXCR4 signaling and downregulation of IL10 (Fig. 10A). Astrocytes in the midbrain also presented a pro-inflammatory state with activation of TNF, IFNG, IL1, IL8, IL17, CD44, CD40 or CXCR4 pathways and inhibition of IL10 or CTLA4 signaling (Fig. 10B). Next, we compared the upstream regulators and biological functions predicted to be modulated in the midbrain of MPTP models and in PD patients. Human and cMPTP CD11b^+^ cells shared most of their upstream regulators and functions related to the activation of the immune system and the unfolded protein response (Fig. 10C). The inflammatory activation state of sMPTP CD11b^+^ cells differed from their counterparts in PD and cMPTP. Common pathways shared by PD and MPTP mice were related to phagocytosis (Fig. 10C). Predicted upstream regulators in human astrocytes showed a good correlation with the cMPTP astrocytes and a poor correlation with sMPTP cells (Fig. 10D). The biological functions with the best *Z*-score in human astrocytes were related to cytoskeleton and morphology. However, they were absent or underscored in the MPTP experimental models (Fig. 10D). The high density of dopaminergic cell bodies in the mouse SNpc compared to the equivalent human region may account for these differences. Human and cMPTP astrocytes shared activated pathways related to immune cell infiltration into the brain, and human and sMPTP astrocytes shared functions related to nuclear factor of activated T cells (NFAT) and IL15 (Fig. 10D). CD11b^+^ and ACSA2^+^ cell activation in the experimental models failed to upregulate pathways related to B lymphocytes that were present in the human samples (Fig. 10C and D). Since human samples correspond to advanced stages of the disease (Braak stages 5–6) it could be expected a higher similarity with the cMPTP model. Therefore, these results validate the cMPTP as a representative model of a late stage PD.

## Discussion

In this study we have identified different glial activation states in response to MPTP-induced degeneration of the nigrostriatal pathway. In the striatum, at early stages of neurodegeneration, microglia exhibited a pro-inflammatory profile that was counteracted by an anti-inflammatory phenotype in astrocytes that resulted in decreased T cell infiltration in the sMPTP mice. However, at the same time, a chronic MPTP exposure induced a mild dopaminergic terminal loss accompanied by a transient CD4 T cell infiltration that suggests a pro-inflammatory environment. In the midbrain, the acute neuronal loss generated in the sMPTP mice provoked opposing glial responses, anti-inflammatory in microglia and pro-inflammatory in astrocytes, that resulted in decreased T cell infiltration. Chronic administration of MPTP induced a progressive degeneration of the nigrostriatal pathway that generated a pro-inflammatory reaction and increased CD4 T cell infiltration. In the striatum, microglia and astrocytes cooperated in the phagocytic removal of dopaminergic terminals at early stages of degeneration, once the degenerative process was chronified, only microglia maintained this activity. In the midbrain, the complementary phagocytic activity of glial cells differed. After intense damage (sMPTP), astrocytes were the main phagocytic mediators while microglia were the major phagocytic cells in the chronic state. Our results show that both MPTP administration regimens lead to dopaminergic degeneration and motor impairment, but the activation state of glial cells and the inflammatory response generated along the process differ. The glial activation pattern in the cMPTP reflects many pathways of their corresponding counterparts in the human brain.

Subacute and chronic administration of MPTP decreases the number of TH-positive neurons in the SNpc [42–46]. Although in some early studies motor impairment with subacute MPTP intoxication was not evident [42, 43], in our hands, mice receiving this administration pattern experienced motor disabilities in the rotarod, bar and pole tests. The MPP^+^, the toxic metabolite of MPTP, leads to dopaminergic death by mechanisms that involve mitochondrial dysfunction and oxidative stress [47]. Even though the mechanism is the same, the intensity of damage caused by each intoxication protocol induces different inflammatory responses in the striatum and in the midbrain. Dopaminergic degeneration caused by subacute MPTP intoxication induces complementary activation patterns in microglia and astrocytes. In this context of intense dopaminergic neuronal loss, midbrain CD11b^+^ cells show an anti-inflammatory response and astrocytes polarize towards a pro-inflammatory activation state in detriment of their homeostatic functions. Under the same conditions, striatal CD11b^+^ cells adopt a pro-inflammatory phenotype while astrocytes become anti-inflammatory. Independently of the cell type involved, the generation of an anti-inflammatory response decreases T cell infiltration. These data are in line with our previous observations about the immunosuppressive and anti-inflammatory profile acquired by midbrain CD11b^+^ cells in response to LPS [8] or α-synuclein-induced dopaminergic degeneration [30].

In an attempt to define microglial activation states under neurodegenerative conditions, different microglial signatures have been described [48]. Classical signatures include MGnD established from different mouse models of neurological diseases including ALS, AD and MS [34] or DAM obtained from an AD and ALS transgenic mouse model [35]. In these experimental models, only striatal CD11b^+^ cells in cMPTP mice show correlation with up- and downregulated genes described in DAM. The rest of CD11b^+^ subsets share downregulated transcripts with DAM, reflecting the loss of homeostatic functions [35] and do not exhibit common features with MGnD. The anti-inflammatory profile of sMPTP midbrain CD11b^+^ cells, and the activation of TLR receptor signaling and TNF-related pathways in astrocytes suggests a direct activation, rather than microglia-mediated, of these cells [2, 27]. Single-cell RNA sequencing of microglial cells isolated from the hippocampus of a mouse with severe neurodegeneration at multiple time points identified disease-specific microglial states [49]. Early states were characterized by proliferation and late states by the immune response [49]. In our experimental approach, data obtained from sMPTP and ecMPTP mice midbrain show an increased number of Iba1^+^ and/or CD11b^+^/Ki67^+^ cells that could reflect an early state that is accompanied by a low phagocytic activity. However, our early activation state induces opposing inflammatory programs which is in line with previous observations [50]. The chronic MPTP mouse model shows a late activation profile that shares the pro-inflammatory phenotype characterized by activation of antigen-presenting pathways, IFN responses (*Ifitm2, Ifitm3, Irf2bp2*) and CD4 T cell infiltration [49] and exhibits greater similarities with the human cells.

Innate responses in the striatum and midbrain are triggered cooperatively by microglia and astrocytes. Degeneration of the nigrostriatal pathway activates a phagocytic program in glial cells to eliminate dopaminergic terminals in the striatum and cell bodies in the midbrain. According to our results, phagocytic activity in microglia is a late event in the midbrain while it is maintained at all stages in the striatum. Regional differences described in rodent and human microglia [50, 51] point to an aberrant clearance phenotype in striatal microglia associated with neuronal-driven impaired motor responses [50]. The gene expression signature that defines this aberrant activity is absent in striatal phagocytic cells of MPTP mice. Phagocytic glial cells gene expression profile in MPTP mice show a clear “eat me” state [52]. Exposure to and clearance of dying cells induce a distinct gene expression that is dissociated from pro-inflammatory responses [50]. Thus, regional context would determine whether the phagocytic activity is accompanied by an inflammatory reaction. Apoptotic cells suppress monocyte production of pro-inflammatory cytokines and induce the expression of the anti-inflammatory cytokines [53]. Visualization of apoptotic cell engulfment is difficult due to the efficiency of the process [54, 55]. Based on the gene expression profile of midbrain microglia, the anti-inflammatory phenotype and the lack of detection of active phagocytic activity, we speculate that these cell cells are resolving neuronal damage effectively. The presence of persisting apoptotic cells, such as in the cMPTP [33] and in PD can activate the innate immune system to release pro-inflammatory mediators [55]. In general, phagocytic functions have been attributed to the ameboid, bushy and hypertrophic microglial morphologies [30, 56, 57]. However, our data indicate that morphological diversity can be attributed to changes in the basal activation state of microglia, but not to specific functions.

Our results indicate a context-dependent cooperativity of microglia/myeloid cells and astrocytes in response to neuronal damage. Novel approaches targeting these cells to control the inflammatory response may have therapeutic potential for the treatment of PD. However, to develop this type of therapies is key to understand the generation of inflammatory responses in the striatum and the midbrain to reach the nigrostriatal pathway, and to select the experimental models that better reflect this aspect of PD.

### Electronic supplementary material

Below is the link to the electronic supplementary material.

Supplementary Material 1

## Data Availability

CD11b^+^ and ACSA2^+^ RNA sequencing data are publicly available in NCBI’s Gene Expression Omnibus (GEO) and are accessible through GEO Series accession number GSE191131. All data are available in the main text and supplementary materials. Any additional information related to this study will be available from the corresponding author (M.A.) upon request.

## Abbreviations

AD: Alzheimer´s disease
ALS: Amyotrophic lateral sclerosis
CB: Cytometry buffer
CI: Confidence Intervals
cMPTP: Chronic MPTP mouse model
DAM: Disease-associated microglia
ecMPTP: Early chronic MPTP mouse model
GEO: Gene Expression Omnibus
GSEA: Gene set enrichment analysis
GWAS: Genome-wide association studies
HLA-DR: Human leukocyte antigen-DR isotype
IFNγ: interferon-γ
IL1β: interleukin 1 beta
IPA: Ingenuity Pathway Analysis
IPD: Idiopathic Parkinson’s disease
LPS: Lipopolysaccharide
MPTP: 1-methyl-4-phenyl-1,2,3,6-tetrahydropyridine
MS: Multiple Sclerosis
*NAc*: *Nucleus accumbens*
NFAT: Nuclear factor of activated T cells
PD: Parkinson’s disease
RNA-seq: RNA-sequencing
*RT*: *Room Temperature*
sMPTP: Subacute MPTP mouse model
SNpc: Substantia nigra pars compacta
snRNA-seq: Single nuclei RNA sequencing
TH: Tyrosine Hydroxylase
TLRs: Toll-like receptors
TNFα: Tumor necrosis factor alpha
VTA: Ventral tegmental area
