# Supplementary material for: Nigrostriatal degeneration determines dynamics of glial inflammatory and phagocytic activity
Source: J Neuroinflammation. 2024 Apr 12;21:92. doi: 10.1186/s12974-024-03091-x (PMC11015575; doi:10.1186/s12974-024-03091-x)
Supplement: Supplementary file 1 — Supplementary Material 1 [file 12974_2024_3091_MOESM1_ESM.docx]

**Supplementary material**

**
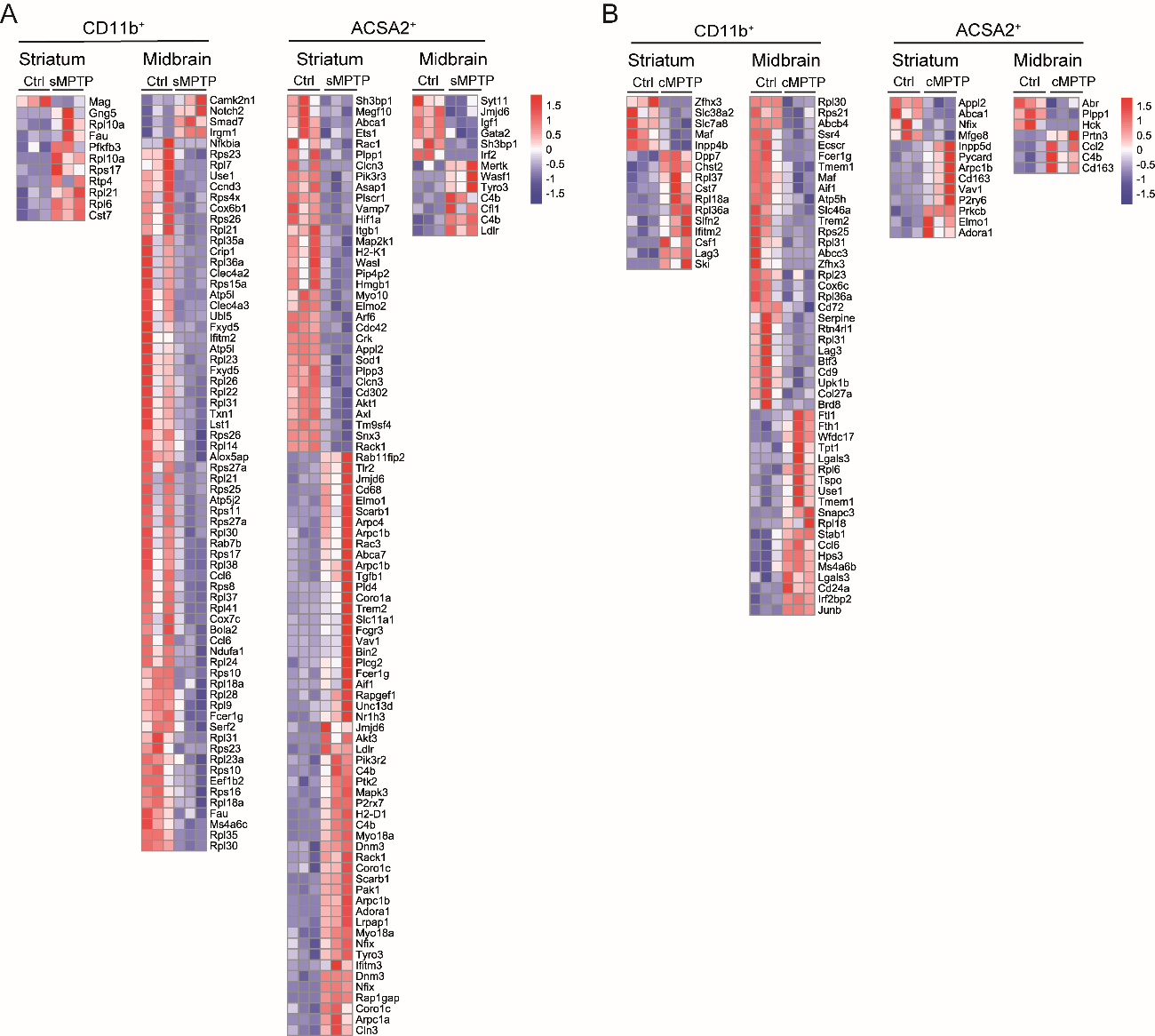
**

**Supplementary figure 1: Heatmaps of differentially displayed phagocytic genes in the striatum and midbrain under parkinsonian conditions.** Differentially displayed genes (*p* < 0.01) present in the phagocytic microglia and in the GO_Phagocytosis (GO0006909) gene data sets were used to generate a specific phagocytic profile of CD11b^+^ and ACSA2^+^ in the striatum and in the midbrain of (A) sMPTP and (B) cMPTP mice. (N = 3 animals/group).

**
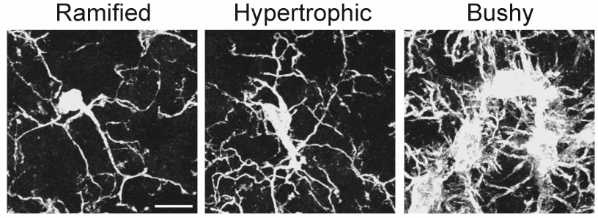
**

**Supplementary figure 2: Iba1^+^ cell classification based on morphology.** Three types of morphologies were selected to assess morphological changes in Iba1^+^ cells. Ramified, which is characterized by a small cell body with long and thin processes. Hypertrophic, characterized by a larger cell body with long and thick processes. Bushy cells have a large cell body and short processes. Magnification bar: 10 µm.

**
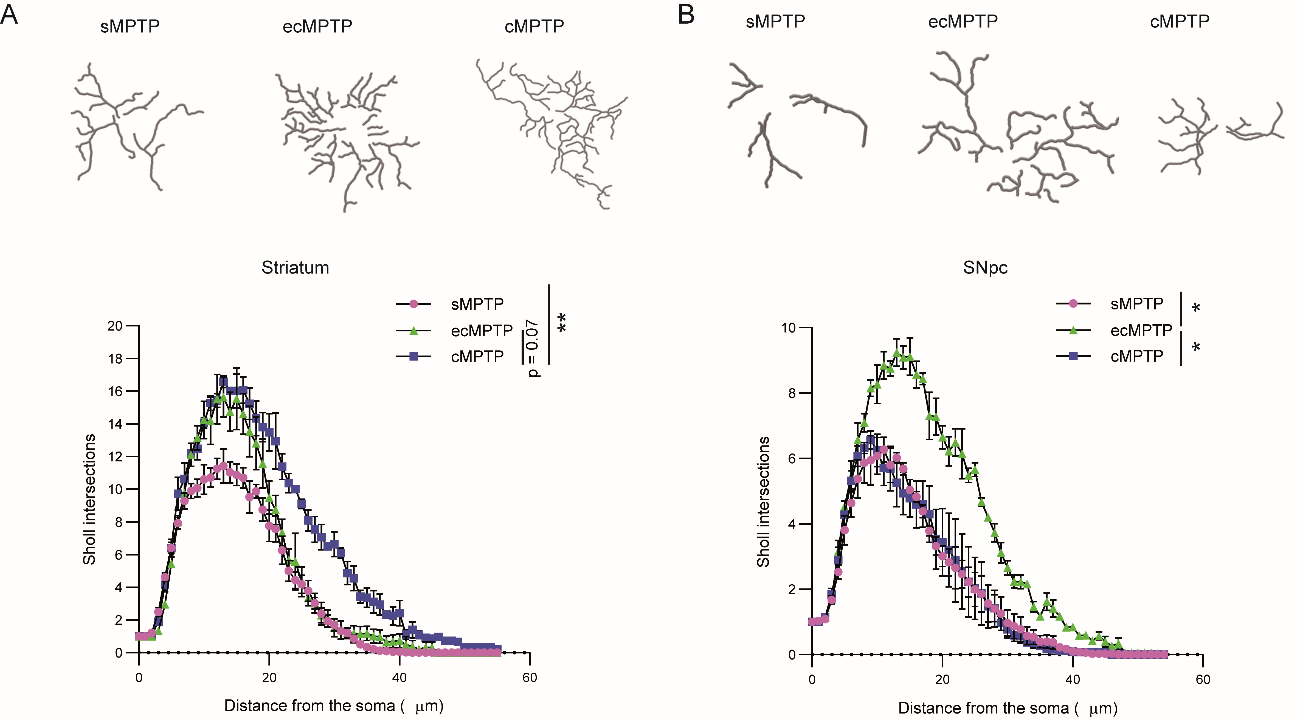
**

**Supplementary figure 3: Ramification of Iba1^+^ cells along MPTP intoxication.** Three-dimensional representations of microglia ramifications and distribution of the number of Sholl intersections as a function of the distance from the microglial soma (intersections per 1 µm) in sMPTP, ecMPTP and cMPTP mice. (A) Sholl analysis in the striatum. (B) Sholl analysis in the SNpc. Data represent the mean ± SEM from 3 animals/group. Statistical analysis: Kruskal-Wallis test followed by Dunn’s multiple comparisons test. **p* < 0.05, ***p* < 0.01.
